# Mapping the cellular etiology of schizophrenia and complex brain phenotypes

**DOI:** 10.1038/s41593-024-01834-w

**Published:** 2025-01-20

**Authors:** Laramie E. Duncan, Tayden Li, Madeleine Salem, Will Li, Leili Mortazavi, Hazal Senturk, Naghmeh Shahverdizadeh, Sam Vesuna, Hanyang Shen, Jong Yoon, Gordon Wang, Jacob Ballon, Longzhi Tan, Brandon Scott Pruett, Brian Knutson, Karl Deisseroth, William J. Giardino

**Affiliations:** 1 Department of Psychiatry and Behavioral Sciences, Stanford University, Stanford, CA USA; 2 Wu Tsai Neurosciences Institute, Stanford University, Stanford, CA USA; 3 Department of Statistics, Stanford University, Stanford, CA USA; 4 Vice Provost for Undergraduate Education, Stanford University, Stanford, CA USA; 5 Department of Psychology, Stanford University, Stanford, CA USA; 6 Department of Computer Science, University of San Francisco, San Francisco, CA USA; 7 Department of Epidemiology, Stanford University, Stanford, CA USA; 8 VA Palo Alto Health Care System, Palo Alto, CA USA; 9 Department of Neurobiology, Stanford University, Stanford, CA USA; 10 School of Medicine, University of Alabama at Birmingham, Birmingham, CA USA; 11 Department of Bioengineering, Stanford University, Stanford, CA USA

**Keywords:** Psychiatric disorders, Genetic association study, Diagnostic markers, Alzheimer's disease

## Abstract

Psychiatric disorders are multifactorial and effective treatments are lacking. Probable contributing factors to the challenges in therapeutic development include the complexity of the human brain and the high polygenicity of psychiatric disorders. Combining well-powered genome-wide and brain-wide genetics and transcriptomics analyses can deepen our understanding of the etiology of psychiatric disorders. Here, we leverage two landmark resources to infer the cell types involved in the etiology of schizophrenia, other psychiatric disorders and informative comparison of brain phenotypes. We found both cortical and subcortical neuronal associations for schizophrenia, bipolar disorder and depression. These cell types included somatostatin interneurons, excitatory neurons from the retrosplenial cortex and eccentric medium spiny-like neurons from the amygdala. In contrast we found T cell and B cell associations with multiple sclerosis and microglial associations with Alzheimer’s disease. We provide a framework for a cell-type-based classification system that can lead to drug repurposing or development opportunities and personalized treatments. This work formalizes a data-driven, cellular and molecular model of complex brain disorders.

## Abstract (web summary)

Duncan and colleagues link specific human brain cell types to schizophrenia and other complex brain phenotypes, providing mechanistic insights and a cellular taxonomy for psychiatric disorders.

## Main

Approximately one in five adults has a psychiatric disorder^1^. While these disorders may resolve on their own or with treatment, for many they represent lifelong afflictions. Among psychiatric disorders, schizophrenia is noted for its severity and for the inadequacy of currently available treatments^2,3^. Like all other major psychiatric disorders (depression, substance use disorders, posttraumatic stress disorder (PTSD)), the etiology of schizophrenia depends both on genetic and environmental factors. Schizophrenia is one of the most heritable of these complex polygenic disorders, with heritability estimates from twin studies of approximately 80%^4,5^.

**Fig. 1:**
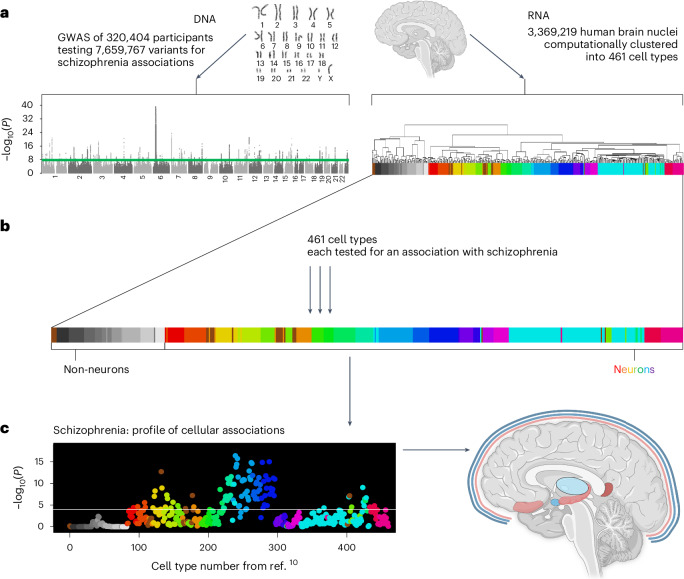
Approach for systematically testing 461 human brain cell types for association with schizophrenia (and other phenotypes tested). We tested whether genes associated with schizophrenia were preferentially expressed in one or more brain cell types using linear regression and Bonferroni correction for 461 tests. **a**, We used two types of human genome-wide data. Left, Results from the most recent schizophrenia GWAS (*n* = 320,404 participants). The GWAS results are depicted using a Manhattan plot with the chromosomal position on the *x* axis and statistical significance (−log_10_(*P*)) on the *y* axis for each genetic variant tested. Right, Cell type data from the most comprehensive human brain snRNA-seq study to date (3,369,219 cells from 105 brain regions, clustered into 461 statistical clusters, referred to as cell types; see Fig. 2 for the color coding). **b**, Each of the 461 cell types was tested for association with schizophrenia using linear regression. As detailed in the Methods, we first calculated ‘specificity’ scores to quantify the fraction of each gene’s total expression found in each cell type. Specificity values ranged from 0 to 1; for each gene the specificity scores summed to 1 over the 461 cell types (by definition). MAGMA was first used to quantify the schizophrenia associations for each gene. Next, for each of the 461 cell types (analyzed separately), we used linear regression (implemented in MAGMA) to test for linear relationships between specificity scores and the schizophrenia associations of those genes, while correcting for known potential confounding variables. **c**, Results are depicted first as a cell type profile (for schizophrenia); then, associated cell types are described with functionally relevant details (for example, brain structure and inferred neurotransmitter use, receptor expression and cortical layer localization). Karyotype in **a** (top left) reproduced with permission from ref. ^11^ under a Creative Commons license CC BY-NC-ND 4.0 and dendrogram lines in **a** (right) reproduced with permission from ref. ^10^, AAAS. Brain image in **a** (top right) created with BioRender.com.

Human genome-wide association studies (GWAS), encompassing millions of individuals, have demonstrated that common genetic variants account for a substantial fraction of population liability to schizophrenia and other psychiatric disorders^6^. For instance, the most recent GWAS of schizophrenia found 287 risk loci in a sample of 320,404 participants (Fig. 1a). These loci exceeded a stringent international threshold for statistical significance (*P* < 5 × 10^−8^), which corrects for testing of multiple hypotheses. While these landmark studies revealed thousands of genetic risk factors for psychiatric disorders, they also raised a new challenge: determining the physiological relevance of associated genomic loci.

Polygenic influences on schizophrenia offer a powerful entry point for discovering disease etiology when combined with data about how genes are used in specific brain cell types. Differential gene expression generates cellular diversity in the body and newly available technology allows the measurement of gene expression (RNA molecule counts from individual cells using snRNA-seq and scRNA-seq for single cells). Moreover, computational clustering of cellular transcriptomes readily reveals known and new cell types; consequently, this approach is used to create comprehensive atlases of cell types in human and animal organs^7–9^. Recently, a landmark snRNA-seq dataset for the human brain was released^10^. Previous human studies surveyed far fewer brain regions (for example, 1–3 brain regions). Thus, the analysis of 105 brain regions and 3,369,219 individual nuclei by Siletti et al.^10^ is the most comprehensive human snRNA-seq dataset available to date. This dataset afforded statistical clustering of 461 cell types in healthy human brains, 378 of which are neuronal. The right side of Fig. 1a, adapted from ref. ^10^, shows these 461 cell types as the ends of the leaves of a dendrogram. Using this dataset, which specifies gene expression in cell types from healthy, adult human brains, we were able to map the genetic risk for schizophrenia^6^ to specific human brain cell types. This was accomplished in an entirely data-driven manner using the leading available genome-wide and brain-wide datasets and is thus unbiased with regard to researcher-driven hypotheses about which cell types should be associated with schizophrenia. Note that supercluster refers to broader groupings of these 461 cell types^10^. The karyotype image in Fig. 1a is from ref. ^11^.

Building on the work of Watanabe et al.^12^, Skene et al.^13^ and Bryois et al.^14^ (who used rodent data and earlier human snRNA-seq datasets), we used the MAGMA^12,15^ software to analyze the schizophrenia GWAS results implementing gene property analysis. We determined the cell types with gene use that was positively correlated with schizophrenia gene associations, while adjusting for potential confounders (for example, gene size and gene density; see the Methods for the details). We then used conditional analyses to empirically select representative cell types among all significant results for further discussion.

The 461 cell types tested in this study are hierarchically organized; numerically close cell types tend to have similar gene expression profiles. This means that there are not 461 statistically independent cell types, and that Bonferroni correction is approximately one order of magnitude too stringent. Nevertheless, we implemented Bonferroni correction to be conservative. This also means that significant cell types may be significant for two reasons: (1) either they are driving a phenotypic association (truly associated); or (2) they appear associated because they share overlapping gene expression with a cell type (or types) driving the associations. The latter scenario (correlated statistical tests) is a common problem relevant to several domains of research. In this study, we used conditional analysis (Methods), a leading statistical approach for handling this issue^13,14^. Interpretation-wise, it is most conservative to only focus on these relatively independent, significant cell types, hence our primary focus on these cell types.

To summarize, we used robust statistical procedures to combine massive, unbiased, genome-wide and brain-wide datasets to systematically test which brain cell types were linked with schizophrenia. To validate this approach, we also analyzed four informative comparison phenotypes (alcohol consumed per week^16^, sleep duration per night^17^, multiple sclerosis^18^ and Alzheimer’s disease^19^). These four phenotypes were critical for demonstrating that this method correctly identified true positives, that is, cell types that are known or strongly suspected to be associated with these phenotypes. We also provide, in the supplementary materials, the results for other psychiatric disorders (depression, bipolar disorder, anorexia, alcohol use disorders, PTSD, autism and attention-deficit/hyperactivity disorder (ADHD); Methods). Throughout this article, please refer to Supplementary Table 1 for complete information about the 461 cell types analyzed in this article (numbered 0–460; ref. ^10^).

Two methodological design choices merit special mention, namely, the choice of analysis method (MAGMA^12,15^) and the choice of phenotypes to analyze. We chose MAGMA because it has been the dominant approach in the field for this type of analysis (GWAS + scRNA-seq/snRNA-seq) and appropriate control of false positives has been repeatedly demonstrated. Furthermore, we used proximity-based MAGMA as our baseline analysis given that functional information is not yet available for the specific cell types analyzed in this study; thus, proximity is a conservative approach that probably captures many (but certainly not all) functional elements. Arguably the strongest contender to MAGMA is the single-cell disease relevance score^20^; however, the primary rationale for this approach (that is, not relying on predefined cell types) is a major limitation for interpretation. Indeed, interpretations typically revert to cell types. The other strong contender is linkage disequilibrium score regression (LDSC) of specifically expressed genes (SEGs)^21^, which we use to illustrate variability in results from different methods (see the ‘Statistics and reproducibility’ section). Second, we intentionally focused on a small number of highly informative phenotypes rather than using the ‘kitchen sink’ approach of analyzing many phenotypes of variable quality. This choice afforded sufficient space to describe schizophrenia results in detail. It was also essential for demonstrating the validity of this approach via the correct identification of expected cell types for our four comparison phenotypes.

## Results

### Overview of schizophrenia-associated cell types

**Fig. 2:**
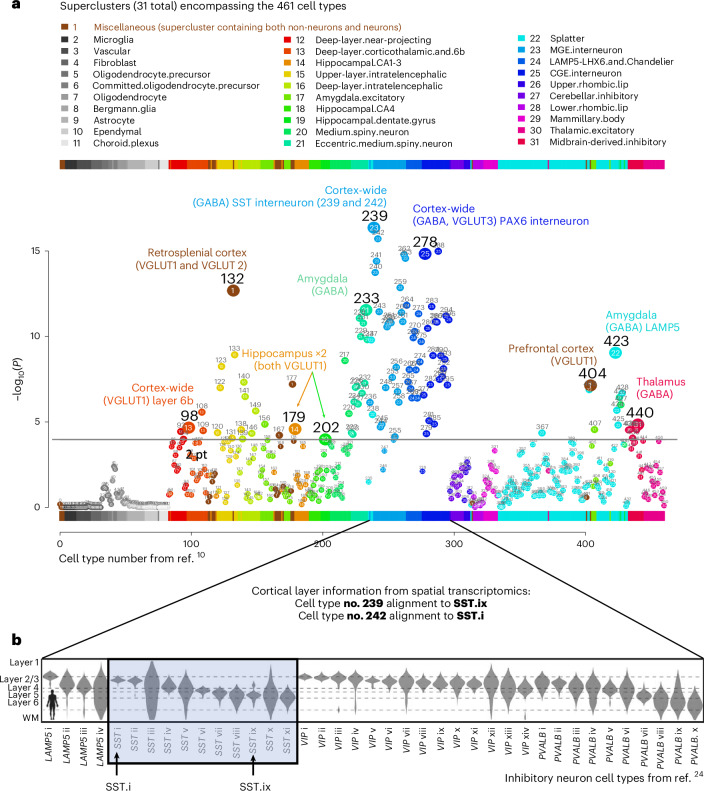
Cell type associations for schizophrenia. **a**, Schizophrenia results for 461 cell types are depicted in a scatterplot, with cell type number on the *x* axis and statistical significance on the *y* axis (−log_10_(*P*)), such that higher values are more statistically significant. The horizontal gray line denotes Bonferroni correction for the 461 regression analyses (*P* < 0.0001). The numbers above the points are cell type numbers; the numbers within the points are supercluster numbers. Larger points denote the ten cell types that were identified as relatively independent using conditional analyses. **b**, The top cell type for schizophrenia (no. 239) aligned to the SST interneuron subtype SST.ix (from ref. ^24^) for which cortical layer localization was determined using spatial transcriptomics. Thus, we inferred that cell type no. 239 was probably most abundant in cortical layer 5, but also found in cortical layers 6 and 2 and 3, whereas cell type no. 242 was probably most abundant in layers 2 and 3. Panel **b** adapted with permission from ref. ^23^, AAAS.

Of the 109 cell types that were significantly associated with schizophrenia, there were ten relatively independent, significant cell types (Fig. 2a). The most significant was a subtype of somatostatin (SST) interneurons (no. 239, *P* = 4.3 × 10^−17^). The next two were also cortical: PAX6 interneurons distributed widely across the cortex (no. 278, *P* = 1.5 × 10^−15^) and an excitatory cell type found almost exclusively in the retrosplenial cortex (no. 132, *P* = 2.1 × 10−^13^, 91% of cells from the retrosplenial cortex); no. 132 is probably from cortical layer 5, as per ref. ^10^. Note that the PAX6 interneurons (no. 278) were annotated as GABA/VGLUT3, indicating coexpression of GABA and glutamate, which is relatively uncommon. Fourth and fifth were two distinct inhibitory amygdala neuron types (no. 233, *P* = 2.8 × 10^−12^ and no. 423 (*P* = 9.0 × 10^−10^)). The remaining five cell types had neurons primarily from the prefrontal cortex (no. 404, *P* = 7.3 × 10^−8^; Brodmann area 14), thalamus (no. 440, *P* = 1.4 × 10^−5^), cortex-wide excitatory neurons annotated to deep layer 6b (no. 98, *P* = 2.2 × 10^−5^) and two excitatory hippocampal neuron types (no. 179 *P* = 2.6 × 10^−5^ and no. 202 *P* = 1.1 × 10^−4^). In addition to these ten relatively independent, significant cell types, other notable significant cell types were medium spiny neurons in the striatum (that is, caudate and putamen, no. 222), cortex-wide excitatory neurons in layer 2/3 (no. 123), and cell types preferentially located in the visual cortex (no. 133), septal nuclei (no. 428), superior colliculus (nos. 433 and 367) and substantia innominata (no. 232). For the full results, see Supplementary Table 1.

### Interneurons associated with schizophrenia match and extend previous findings

For schizophrenia, arguably the most widely replicated postmortem tissue finding involves aberrant inhibitory neurons in the cortex (interneurons)^22,23^. While both the parvalbumin and SST subclasses of interneurons have been implicated, the most recent evidence showed the greatest abnormalities in SST interneurons^22,23^. Thus, the present report of the strongest association with SST interneurons, and many significantly associated interneurons (including parvalbumin), is concordant with leading previous findings. We extended these findings by specifying more subtle subtypes of SST interneurons and by determining the probable cortical layers for these schizophrenia-associated SST interneuron subtypes (nos. 239 and 242) using additional snRNA-seq and spatial transcriptomics datasets. We also examined cell type no. 242 because it was nearly as significant as the top cell type (no. 239). This second-most significant cell type (no. 242) was also associated with other psychiatric phenotypes, included ~3× more cells than no. 239 and it was the most significant cell type in our analysis of the 2018 schizophrenia GWAS. Thus, we thought no. 242 was also a strong candidate for psychiatric relevance.

Recent studies identified far more interneuron subtypes than were distinguishable using traditional markers. These transcriptomically defined cell types reside preferentially in specific cortical layers in both humans and mice^24^. Figure 2b, using a plot adapted from ref. ^24^, shows the cortical layer distribution of human interneuron subtypes (that is, cortical layers 1–6). We aligned SST subtypes and found that nos. 239 and 242 (from ref. ^10^) best matched SST.ix and SST.i, respectively (from ref. ^24^), and that these cell types differed in cortical layer distribution. No. 239 SST interneurons were most likely to be localized to cortical layer 5 (less so to layers 6 and 2/3), while no. 242 SST interneurons were most likely to be in layers 2/3. Alignment to two mouse datasets^25,26^ yielded similar cortical layer localization results, providing additional support for putative layer localizations. Contextualizing these findings, we note that schizophrenia postmortem tissue studies have primarily identified deficits in upper cortical layers, particularly deep layer 3 (ref. ^27^), which may be consistent with the primary localization of cell type no. 242 and, to a lesser extent, no. 239. Given known disruptions in sensory processing and integration in schizophrenia, it is conceivable that cortical layer 5 integration and output functions^28^ are disrupted in schizophrenia because of abnormalities in layer 5 SST interneurons of the no. 239 type.

### Subcortical neuronal associations with schizophrenia

In the amygdala, a structure known to have diminished volume in schizophrenia^29^, we found 17 significant cell types (including both inhibitory and excitatory), two of which were independent significant cell types (nos. 233 and 423, both inhibitory). Cell type no. 233 was annotated by Siletti et al.^10^ as a medium spiny neuron of the eccentric subtype, a recently discovered subtype of medium spiny neurons^9^. This general class of neurons—medium spiny neurons—has been frequently linked to schizophrenia; medium spiny neurons are the dominant inhibitory neuron of the striatum (caudate and putamen). In addition to the two main subtypes of medium spiny neurons, which are well characterized based on their differential expression of dopamine receptors (D1 versus D2), recent transcriptomic studies made it clear that a third subtype of medium spiny neurons also exists. These ‘eccentric’ medium spiny neurons evaded detection because classical D1 and D2 markers do not reliably differentiate the newly named ‘eccentric’ medium spiny neurons^9^. Thus, linking this eccentric medium spiny neuron type (no. 233) to schizophrenia demonstrates the multiple benefits of large-scale transcriptomic studies and the unbiased approaches used in this study to link cell types to schizophrenia. First, scRNA-seq studies made possible the detection of the relatively rare (~4%) but clearly transcriptomically distinct eccentric medium spiny neuron subtype^9^. Second, the brain-wide snRNA-seq dataset from ref. ^10^ used in this study shows that neurons that transcriptomically resemble all three subtypes of medium spiny neurons (D1, D2 and eccentric) are also found outside the striatum. Third, this approach links these newly discovered, relatively rare and extrastriatal eccentric medium spiny neurons to schizophrenia. Such a discovery would not have been possible without these large, comprehensive and unbiased datasets that allow us to see more clearly the gaps in previous knowledge and to discover which newly filled gaps might also provide critical information about schizophrenia etiology. Consistent with previous research, we found striatal associations of medium spiny neurons (nos. 222 and 224).

The second relatively independent, significant amygdala cell type (no. 423) is inhibitory; while found predominately in the amygdala (51% of cells), it is also present in the thalamus (20%), hypothalamus (15%) and other brain regions. Other amygdala cell types associated with schizophrenia, but not deemed independent after conditional analyses, include the medium spiny neurons of the D1 (for example, no. 220) and D2 (no. 217) subtypes.

Our hippocampal findings provide molecular details that can augment and extend schizophrenia imaging and postmortem tissue findings. In this study, we identified specific neuron types that may underlie these hippocampal volume reductions in schizophrenia. Of the 86 neuron types located primarily in the hippocampus (using data from ref. ^10^), seven were significantly associated with schizophrenia in our analyses. Of these, conditional analyses found two relatively independent cell types (nos. 179 and 202). Cell type no. 179 is an excitatory neuron type (VGLUT1); contributing cells came from all major subregions of the hippocampus. Cell type no. 202 is also excitatory (VGLUT1); contributing dissections include all major hippocampal subregions except the subiculum. The present findings may be concordant with previous reports of increased hippocampal excitatory (glutamate) neurotransmitter metabolites in individuals with schizophrenia^30^.

In summary, the primary anatomical locations for the relatively independent, significant schizophrenia cell types were three widely distributed across the cortex, two from specific cortical regions (retrosplenial and prefrontal), two from the amygdala, two from the hippocampus and one from the thalamus. The three subcortical structures linked to schizophrenia in this study (amygdala, hippocampus and thalamus) are precisely the same subcortical structures with the largest volume decreases in patients with schizophrenia compared to controls (Hedges *g* of −0.66, −0.46 and −0.31, respectively) according to a meta-analysis of brain volume studies in first-episode psychosis^29^. Thus, two independent, brain-wide, data-driven approaches to understanding schizophrenia—one using imaging data^29^ and the present approach using genetic data—pointed to the same three subcortical regions: amygdala, hippocampus and thalamus. In the next section, we consider the cell types and brain regions linked to four other phenotypes.

### Contrasting cellular associations for five brain phenotypes

**Fig. 3:**
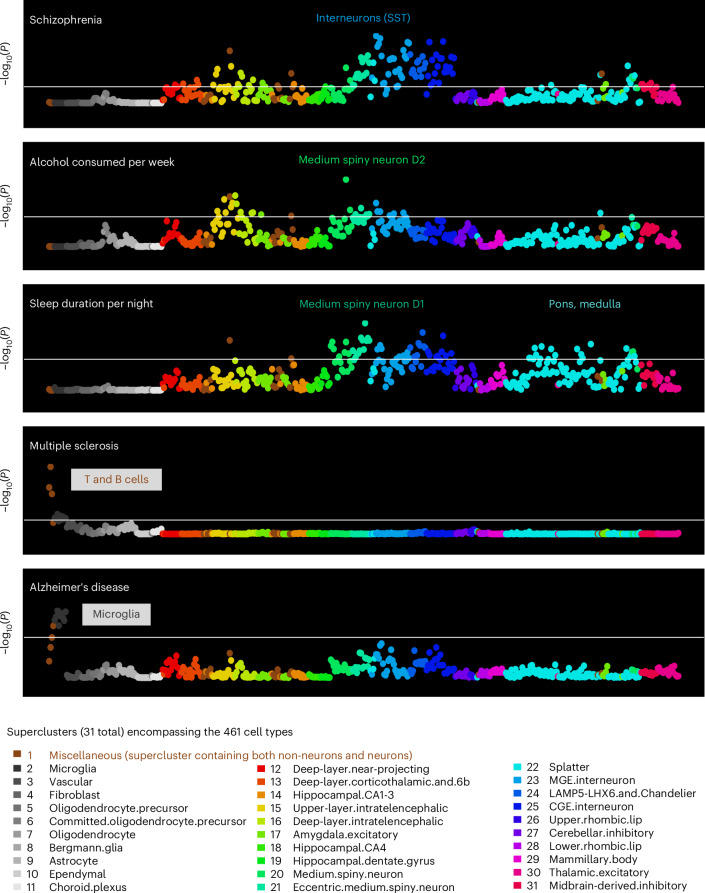
Distinct cell type profiles for five phenotypes. Cell-type associations for five brain-related phenotypes depicted in scatterplots, with cell types depicted in order (for 461 cell types) along the *x* axis and with statistical significance on the *y* axis (reported as −log_10_(*P*), such that higher values are more significant). The horizontal white lines represent the Bonferroni correction for 461 regression analyses (that is, *P* < 0.0001). The first three phenotypes (schizophrenia, alcohol consumed per week and sleep duration per night) yielded neuronal cell type associations. The two neurological phenotypes (multiple sclerosis and Alzheimer’s disease) yielded nonneuronal cell type associations of immune and microglial cell types, respectively. See Extended Data Figs. 1–4 for larger annotated scatterplots that compare the phenotypes.

**Extended Data Fig. 1:**
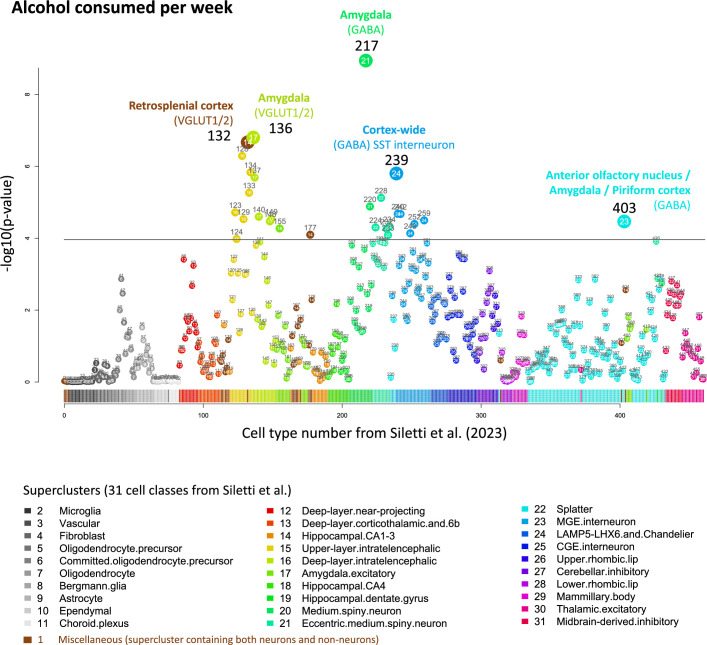
Alcoholic drinks consumed per week cell type associations using Saunders et al.^16^ GWAS. Cell-type associations are depicted in scatterplots, with cell types depicted in order (for 461 cell types) along the x-axis and with statistical significance on the y-axis (reported as -log10(*p*), such that higher values are more significant). Horizontal gray lines reflect Bonferroni correction for 461 regression analyses (that is *p* < 0.0001).

**Extended Data Fig. 2:**
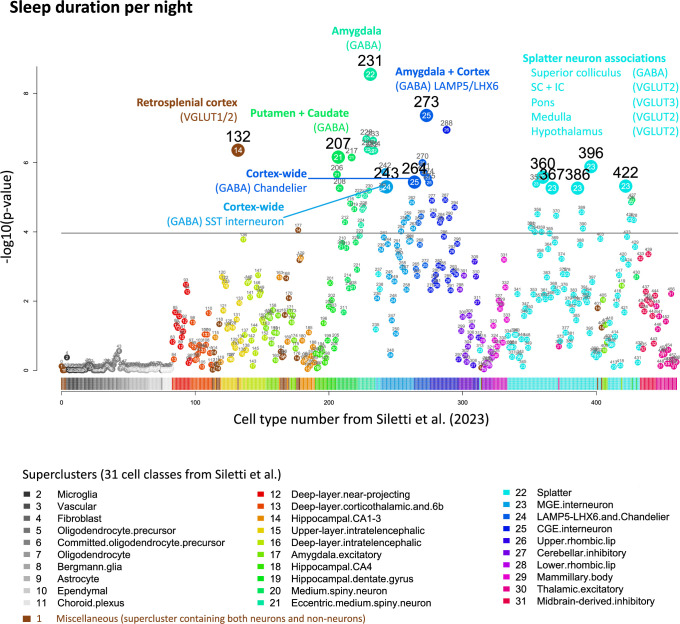
Sleep duration per night cell type associations using Dashti et al.^17^ GWAS. Cell-type associations are depicted in scatterplots, with cell types depicted in order (for 461 cell types) along the x-axis and with statistical significance on the y-axis (reported as -log10(*p*), such that higher values are more significant). Horizontal gray lines reflect Bonferroni correction for 461 regression analyses (that is *p* < 0.0001). Note that the locations of splatter neuron cell types are listed in ascending order: #360, #367, #386, #396, #422; corresponding to superior colliculus, superior + inferior colliculus (SC + IC), pons, medulla, and hypothalamus.

**Extended Data Fig. 3:**
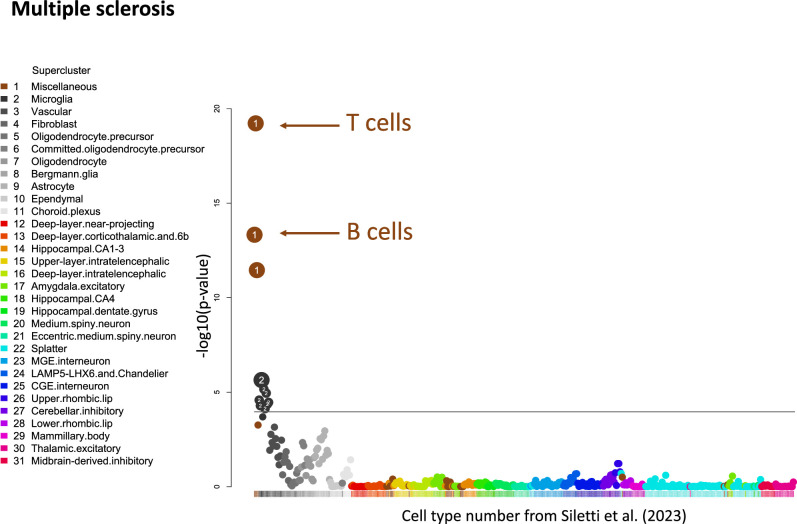
Multiple sclerosis cell type associations using International Multiple Sclerosis Genetics Consortium^18^ GWAS. Cell-type associations are depicted in scatterplots, with cell types depicted in order (for 461 cell types) along the x-axis and with statistical significance on the y-axis (reported as -log10(*p*), such that higher values are more significant). Horizontal gray lines reflect Bonferroni correction for 461 regression analyses (that is *p* < 0.0001).

**Extended Data Fig. 4:**
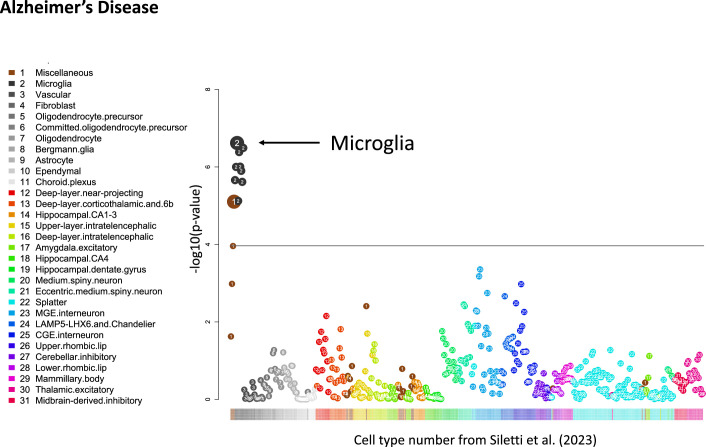
Alzheimer’s disease cell type associations using Bellenguez et al.^19^ GWAS. Cell-type associations are depicted in scatterplots, with cell types depicted in order (for 461 cell types) along the x-axis and with statistical significance on the y-axis (reported as -log10(*p*), such that higher values are more significant). Horizontal gray lines reflect Bonferroni correction for 461 regression analyses (that is *p* < 0.0001).

We sought to establish whether this approach could accurately link cell types to informative complex brain phenotypes: alcohol consumed per week; sleep duration per night; multiple sclerosis; and Alzheimer’s disease. These phenotypes were selected for the presence of expected cellular associations and for the existence of adequately powered GWAS for these phenotypes. Figure 3 shows that the expected cell types were significantly associated with these phenotypes (see Supplementary Tables 2–5 for the full cell type results, Extended Data Figs. 1–4 for the larger annotated scatterplots that compare the phenotypes and Supplementary Tables 6–10 for the gene-level results).

**Fig. 4:**
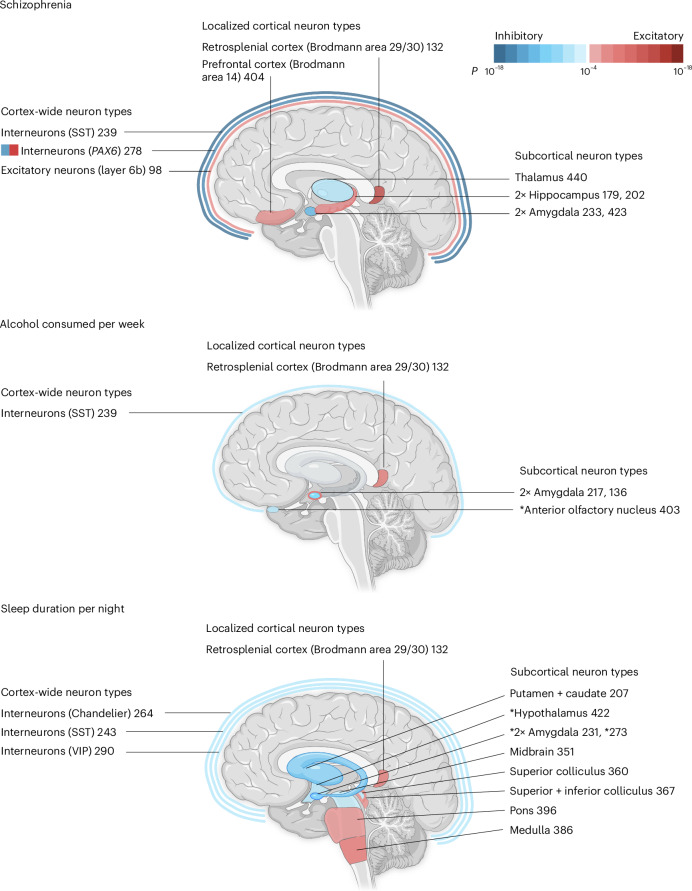
Brain locales of origin for the relatively independent significant cell types associated with schizophrenia, alcohol consumed per week and sleep duration per night. Inhibitory cell types are depicted in blue, excitatory cell types are depicted in red, with color saturation denoting statistical significance from the regression analyses. Unless an asterisk is present, the regions depicted were the source of more than 50% (and usually much more than 50%) of cells for a particular cell type. If there are more than one excitatory or inhibitory cell types for a single brain structure, color saturation corresponds to the more significant cell type. If both excitatory and inhibitory cell types are associated with a single structure, then the fill of the structure (as opposed to the border) denotes the more significant association. For example, there are two amygdala cell types associated with alcohol per week and the inhibitory cell type no. 217 is more significant (hence the blue/inhibitory fill). Cell type no. 278 is annotated as GABA/VGLUT3. VIP, vasoactive intestinal peptide. Figure created with BioRender.com.

For alcohol consumed per week, the top cell type was a D2 medium spiny neuron (no. 217, *P* = 1.3 × 10^−9^), which matched previous expectations given that D2 medium spiny neurons causally influence alcohol consumption^31–35^. For sleep duration per night, the top cell type was also a medium spiny neuron, but of the D1 type (no. 231, *P* = 2.8 × 10^−9^). This finding may be consistent with recent findings linking D1 medium spiny neurons and sleep, particularly rapid eye movement sleep^36^. For sleep duration per night, we also highlight associated cell types from the pons (no. 396, *P* = 1.3 × 10^−6^) and medulla (no. 386, *P* = 5.9 × 10^−6^) as these structures are key nodes of sleep regulatory circuits. Note that these pons and medulla cell types were not associated with schizophrenia, alcohol consumption nor any other psychiatric phenotypes tested; rather, they are specific among these phenotypes for sleep. The sleep phenotype had many associations with the newly named Splatter supercluster of neurons (described in ref. ^10^), which includes a wide variety of subcortical neuron types (see Fig. 4 for a comparison of the brain regions harboring the relatively independent, significant cell types for these three phenotypes).

For multiple sclerosis, an autoimmune condition, the strongest association was a T cell type (no. 1, *P* = 6.0 × 10^−20^). The next most significant cell types were B cells (no. 0, *P* = 4.7 × 10^−14^) and natural killer cells (no. 2, *P* = 3.5 × 10^−12^). These results are consistent with longstanding understanding of pathogenic T cell involvement in multiple sclerosis, the best-available treatments for multiple sclerosis and also more recent findings linking B cells to the disease^37–39^. For Alzheimer’s disease, the most significant cell types were microglial (most associated cell type: no. 6, *P* = 2.4 × 10^−7^). Previous work established that many Alzheimer’s disease genes are preferentially expressed in microglia; newer therapeutics targeting microglia are being investigated^40,41^.

### Data and analytical requirements for sufficient statistical power

**Fig. 5:**
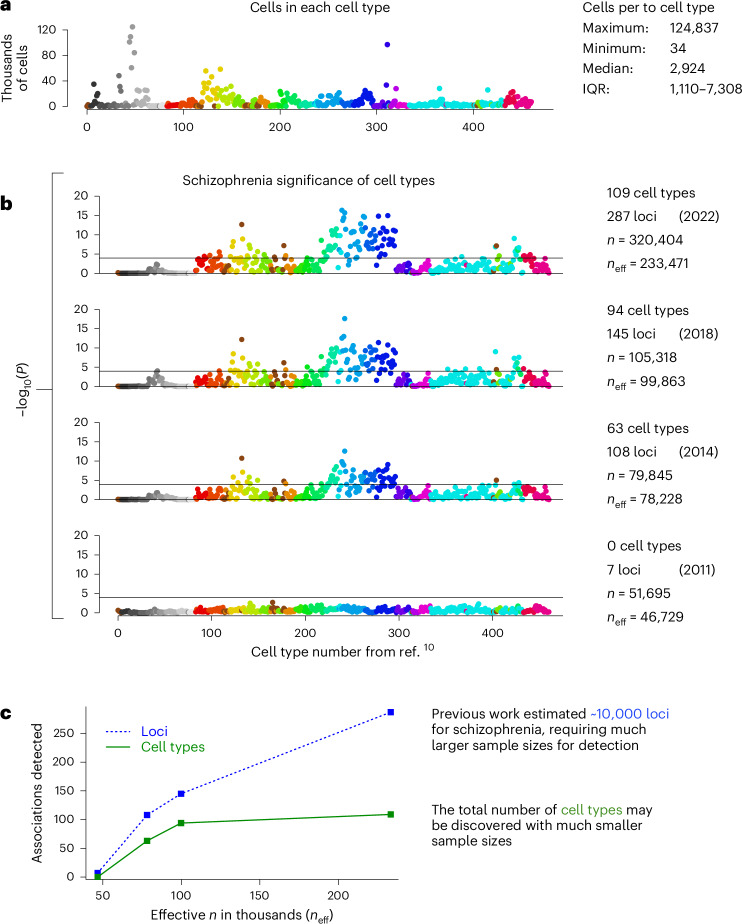
Required statistical power of a GWAS for detecting cell type associations. **a**, Number of cells in each cell type. **b**, Results for four successive, increasingly large and better powered schizophrenia GWAS. The horizontal lines in all plots denote the Bonferroni-corrected statistical significance for 461 regression analyses (*P* < 0.0001). **c**, In this range of sample sizes for schizophrenia GWAS, the number of loci detected continued to increase steeply, but the number of cell types plateaued. Effective *n* accounts for the decrease in statistical power attributable to imbalanced case control ratios: *n*_eff_ = 4/((1/cases) + (1/controls)). IQR, interquartile range.

We sought to determine how the statistical power of individual GWAS influenced our detection of cell type associations, hypothesizing that a poorly powered GWAS would not afford discovery of cellular associations. We further hypothesized that successive GWAS with increasing statistical power (as evidenced by the increasing numbers of loci detected) would afford increasing numbers of associated cell types until most of the relevant cell types were associated, at which time the results would appear to plateau (that is, diminishing discovery of cell types as sample sizes continued to increase). Thus, we reran our primary analysis on four schizophrenia GWAS with progressively increasing sample sizes (and statistical power), ranging from a 2011 GWAS (*n* = 51,695, effective *n* = 46,729) to the most recent GWAS (*n* = 320,404, effective *n* = 233,471)^6,42–44^. Figure 5 shows results that are consistent with our hypotheses. The smallest GWAS revealed no significant cell type associations in our analysis, although it was adequately powered to detect seven GWAS loci. By contrast, the subsequent schizophrenia GWAS afforded detection of 63, 94 and then 109 cell types, corresponding to the discovery of 108, 145 and then 287 loci as reported in primary publications^6,42,43^. Our results suggest that plateauing of cell type discovery may occur at much smaller sample sizes than required for GWAS loci (predicted to saturate at more than 10,000 associated variants for psychiatric disorders). This also suggests that relevant biological features are being captured at the cell-type-level, via aggregation of polygenic signal into cell types.

**Extended Data Fig. 5:**
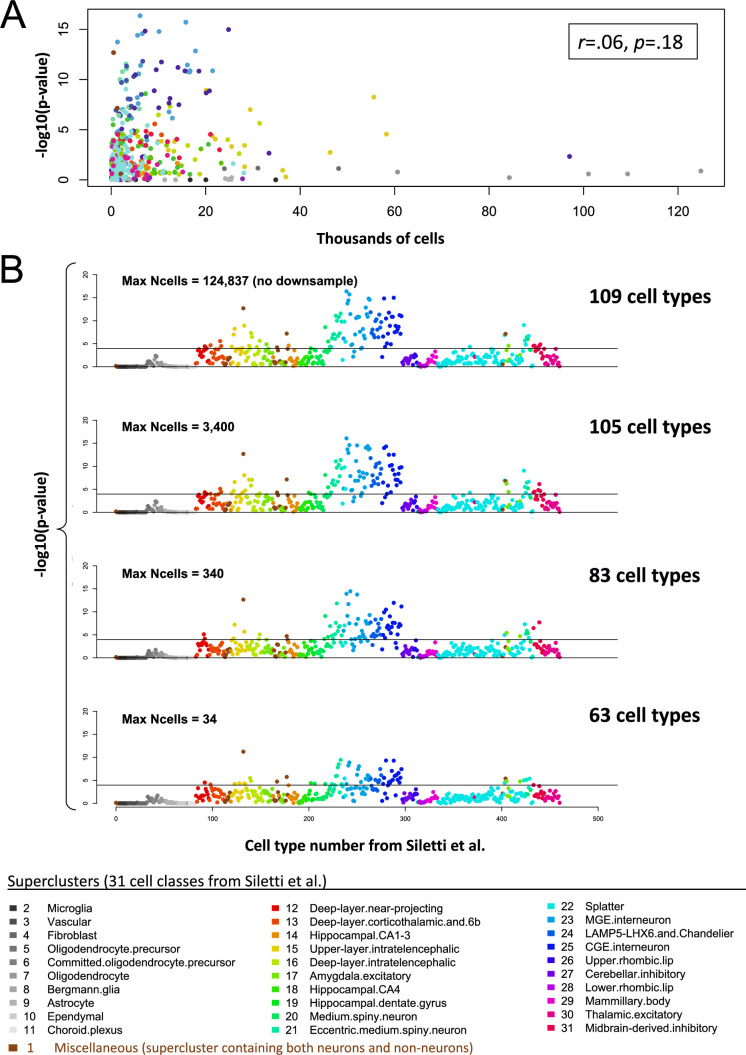
Assessment of the effect of number of cells, in each cell type, on the significance of schizophrenia associations. **A**. There is no systematic relationship between the significance of the schizophrenia association for each cell type (y-axis*)* with the number of cells in the cell type (x-axis); *r* = .06, *p* = .18; from Pearson correlation. **B**. Down-sampling of cells within cell types to a maximum of 3,400, 340, and 34 cells decreased power to detect schizophrenia associations. However, most of the schizophrenia associations detected with no down-sampling (top plot) were detected in all of the down-sampled analyses, even with only 34 cells per cell type (bottom plot).

The statistical power for these analyses also depends on the accuracy of gene expression measured in individual cell types. Cell types with lower numbers of cells tend to have less accurate values for average gene expression. Thus, we first tested whether statistical significance was associated with the number of cells, and it was not (*r* = 0.06, *P* = 0.18 for the correlation between schizophrenia (−log_10_(*P*)) and the number of cells) (see Extended Data Fig. 5a for the graphical depiction). Considering the wide range of cells per cell type (minimum = 34, maximum = 124,837), we also progressively downsampled to assess the effect of lower numbers of cells, in individual cell types, on the power to detect associations with schizophrenia (that is, random sampling of cells in each cell type). Based on the minimum number of cells in any cell type (*n*_cells_ = 34), we downsampled three times to 3,400, 340 and 34 cells (these are maximum values given that some cell types did not have 340 or 3,400 cells). Extended Data Fig. 5b shows that the pattern of associated results was relatively stable across downsampling analyses. Indeed, 56 of the original 109 cell types were significant even when downsampling to just 34 cells per cell type.

We also sought to understand the influence of method-dependent factors on our results. The LDSC-SEG^21^ results are given in Supplementary Table 23. We observed reasonably high consistency between our primary analysis method (MAGMA gene property analysis) and LDSC-SEG: Spearman’s rank correlation for schizophrenia (*r* = 0.71), alcohol per week (*r* = 0.62) and sleep duration per night (*r* = 0.72). However, differences also existed. LDSC-SEG identified no significant cell types for MS despite the established involvement of immune cells; similarly no cell types were associated with AD. The LDSC-SEG analysis also failed to identify D2 medium spiny neurons for alcohol consumption and the pons and medulla cell types for sleep duration per night. The results suggest that LDSC-SEG may not be as well powered as the MAGMA gene property analysis.

### Progress toward a data-driven cellular taxonomy for psychiatric disorders

**Fig. 6:**
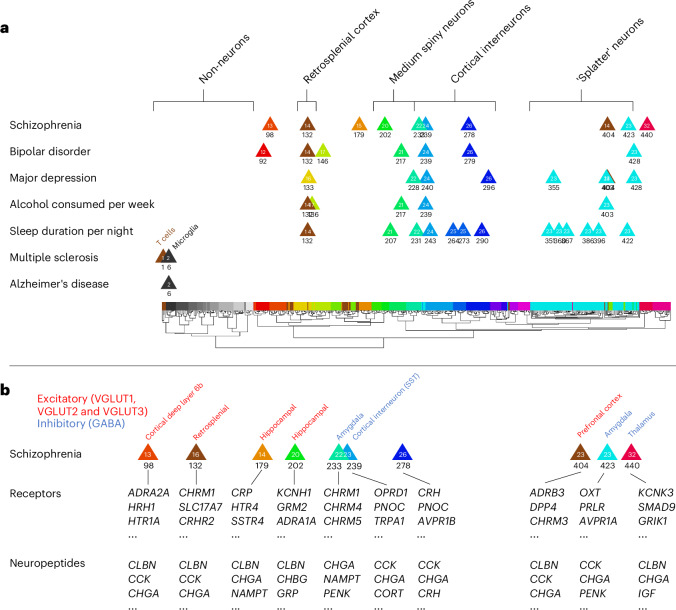
A cellular taxonomy of brain phenotypes maps shared and non-shared cellular associations in a mechanistically informative manner. The 31 superclusters are color-coded as in Fig. 2. **a**, Relatively independent significant cell types are depicted for the five primary phenotypes plus bipolar disorder and depression. Shared or ‘pleiotropic’ cell types include cortical interneurons (no. 239) and excitatory retrosplenial cortex neurons (no. 132). Regarding non-shared cell types, sleep-specific associations of pons (no. 396) and medulla (no. 386) splatter neurons are notable. **b**, Experimental follow-up may include testing of drugs as predicted by gene expression in associated neuron types. ^1^Selected receptor genes among the 200 genes with highest specificity values, for a given cell type. ^2^Selected neuropeptide autoannotation results from ref. ^10^.

**Extended Data Fig. 6:**
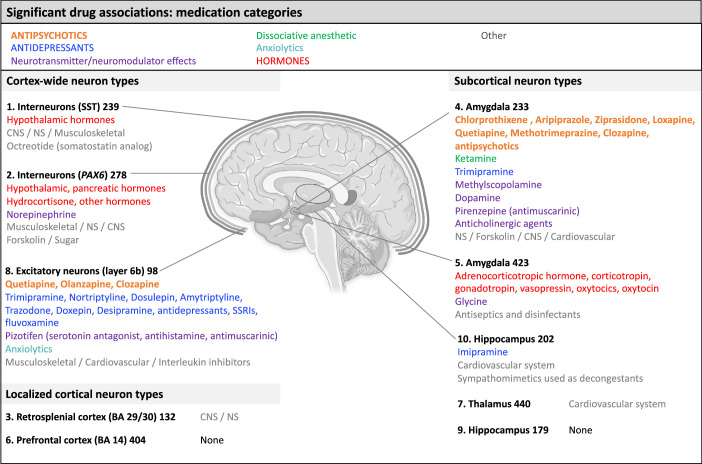
Significant drug-cell type associations, from *in silico* analyses, for the ten relatively independent significant cell types for schizophrenia. Only significant (after FDR-adjustment) results are shown, with a maximum of 20 results per cell type. This figure shows how different schizophrenia-associated cell types might be differentially affected by medications. Of note, only two of the 10 cell types (#233 and #98) had antipsychotic medications among the significant drug results. Antidepressants were among the significant drug results for three cell types (#233, #98, and #202). Ketamine was the second most significant drug association for one cell type (#233) after an antipsychotic medication (chlorprothixene). Hormones were among the significant results for three cell types including the most significant cell type for schizophrenia (#239, hypothalamic hormones). Four cell types had no significant associations with specific drugs (#132, #404, #440, and #179). CNS=central nervous system, NS=nervous system. Figure created with BioRender.com.

This work suggests the possibility of a taxonomy for psychiatric disorders based on quantitative evaluation of cell types (Fig. 6a). A cellular taxonomy could be valuable for structuring future exploration; for example, we can infer biological characteristics of each cell type from transcriptomic information. Figure 6b shows receptor and neuropeptide usage as inferred from transcriptomic data. Further, Extended Data Fig. 6 depicts an in silico exploration of predicted drug-cell type pairings, meaning cell types that putatively are influenced by specific drugs. We also sought to characterize the biological processes, cellular components, and molecular functions of individual cell types via overrepresentation analysis of Gene Ontology (GO) categories (see Methods for details and Supplementary Tables 18–22 for results).

### Extension to other psychiatric phenotypes

**Extended Data Fig. 7:**
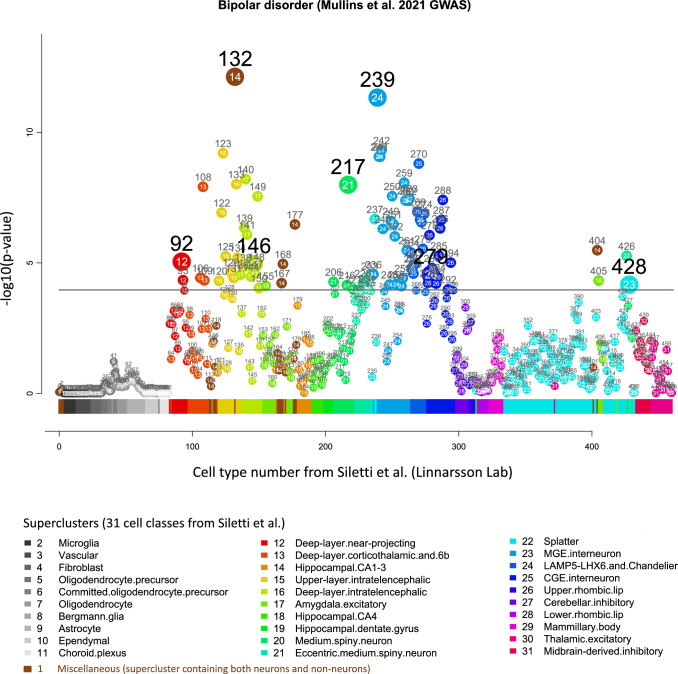
Bipolar disorder cell type associations using Mullins et al.^49^ bipolar disorder GWAS. Cell-type associations are depicted in scatterplots, with cell types depicted in order (for 461 cell types) along the x-axis and with statistical significance on the y-axis (reported as -log10(*p*), such that higher values are more significant). Horizontal gray lines reflect Bonferroni correction for 461 regression analyses (that is *p* < 0.0001).

**Extended Data Fig. 8:**
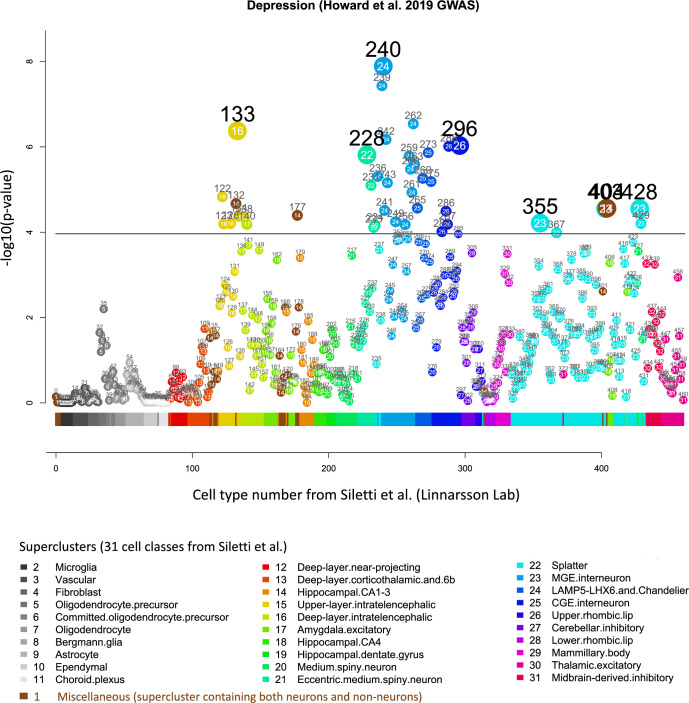
Depression cell type results using Howard et al.^48^ GWAS. Cell-type associations are depicted in scatterplots, with cell types depicted in order (for 461 cell types) along the x-axis and with statistical significance on the y-axis (reported as -log10(*p*), such that higher values are more significant). Horizontal gray lines reflect Bonferroni correction for 461 regression analyses (that is *p* < 0.0001).

**Extended Data Fig. 9:**
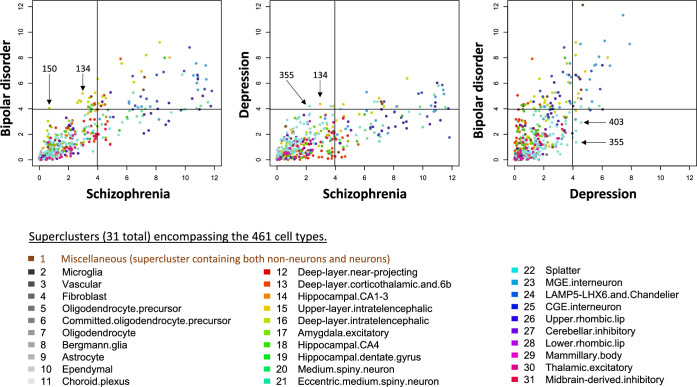
Pair-wise comparison of cell type results for schizophrenia, bipolar disorder, and depression. Axes represent -log_10_(p-value). The upper right quadrants of each plot depict the cell types that are significant for both disorders. Effect size estimates for each of the 461 cell types and standard deviations are available in corresponding Supplementary Tables.

Regarding other psychiatric disorders, bipolar disorder and depression cell types overlapped, but not entirely, with schizophrenia cell types (Fig. 6, Supplementary Tables 11 and 12 and Extended Data Figs. 7–9). Regarding interpretation, it is important to note that published schizophrenia GWAS identified more significant loci and afforded detection of more cell types (109 cell types) than any other psychiatric disorder in this study, including bipolar disorder (83 cell types) and depression (41 cell types). Further, genetic correlations and polygenic scores suggest that phenotypic heterogeneity is probably higher in bipolar disorder and depression datasets than with schizophrenia. Consequently, cell types for bipolar disorder and depression may be less accurate than for schizophrenia because of a variety of factors, including statistical power, true differences in phenotypic heterogeneity and differences in phenotyping quality across phenotypes, thus requiring further study. For other psychiatric disorders, only a few cell types (or no cell types) exceeded statistical significance (*P* < 0.0001). This is likely because of insufficient power in the currently available GWAS for anorexia (no cell types), substance use (no cell types), PTSD (no cell types), autism (no. 428) and ADHD (cell types nos. 136, 234, 377 and 228), and probably not because of a lack of truly associated cell types (for the full results, see Supplementary Tables 13–17).

## Discussion

Major scientific advances often depend on newly available data obtained either in greater detail or scale, or via combined datasets capable of yielding new insights. The vision and perseverance of leaders of the Psychiatric Genomics Consortium enabled the discovery of thousands of specific risk loci for schizophrenia and other psychiatric disorders using a considerably large sample sizes (>1 million participants)^6,16,42,45^. Combining the schizophrenia GWAS results^6^ with another landmark data resource, an snRNA-seq dataset of 3,369,219 unique cells from 105 human brain regions^10^, we were able to discover cell types probably involved in the etiology of schizophrenia (based on their expression of schizophrenia-associated genes). The cell types reported in this study include representatives from the broader neuron types previously associated with schizophrenia (pyramidal neurons, medium spiny neurons and interneurons)^6^.

The more comprehensive snRNA-seq dataset used in this study afforded the detection of more nuanced cell types than previously possible. Highlighting two examples, fear and the sense of self are two symptom domains that are clinically important for many patients with schizophrenia. Indeed, fear is one of the defining emotional features for many who suffer from schizophrenia and the amygdala is critical for fear processing. Thus, the amygdala cell types reported in this study (nos. 233 and 423) may be causally linked to the maladaptive fear that causes so many problems for individuals with schizophrenia. A more amorphous but nevertheless clinically relevant and historically notable feature of schizophrenia concerns distortions in the sense of self. Consequently, it is noteworthy that we discovered a highly associated cell type (no. 132) in a brain region (the retrosplenial cortex) that is critical for the sense of self. Specifically, recent findings showed that a particular rhythm in the retrosplenial cortex causes dissociation^46^, meaning alterations in one’s sense of self. Thus, it is interesting that we found neurons in a brain region critical for an integrated sense of self, given that schizophrenia is a disorder noted for alterations in the sense of self. Moreover, this retrosplenial neuron type is significantly associated with many psychiatric phenotypes and may thus represent a common node of dysfunction in psychiatric disorders.

Historically, classifying psychiatric disorders has proven challenging, generating enduring debate about psychiatric nosology. Our final figure depicts the cell type profiles for the psychiatric and neurological phenotypes analyzed in this study, providing a proto-taxonomy for these phenotypes. We suggest that cell typologies may prove a useful level of analysis for a stable taxonomy of brain disorders, making available the appropriate measurements and organizing principles. What remains to be determined is the relevance of developmental versus adult cell types to different disorders, the identifiability of subtypes of disorders with this approach and the eventual impact of this research on the classification of psychiatric disorders.

Appealingly, the long-postulated heterogeneity in psychiatric disorders may be wrangled into comprehensibility with these approaches. Different cell type associations might be identified, for example, for the major symptom domains of schizophrenia, including positive, negative and disorganized symptoms. Furthermore, by specifying the molecular properties of associated cell types (for example, receptors and neurotransmitters), this classification system may nominate appropriate treatments. By analogy, quantifying and organizing electrons and protons allowed the classification of elements into the periodic table and thus afforded predictions about elemental interactions. A cell typology for psychiatric disorders may similarly afford accurate predictions about which medications and treatments are most likely to be effective for each disorder.

There are important limitations to this approach and currently available datasets. First, this approach cannot discover cellular abnormalities resulting exclusively from environmental influences. Conversely, this limitation also implies that reported cellular associations do not result from environmental risk factors, treatment or the consequences of living with schizophrenia. Second, snRNA-seq and related datasets are still incomplete. Briefly, this snRNA-seq dataset^10^ did not include all brain regions, females or individuals with non-European ancestry. Specific brain regions not sampled in ref. ^10^, which may be particularly important for schizophrenia, include the ventral tegmental area and portions of the dorsolateral prefrontal cortex (Brodmann area 9; Brodmann area 46 is included). Another important next step will be conducting the same analyses using developmental datasets. The statistical methods used in this study were selected for their robustness to false positives and previous use with transcriptomic datasets. Such models may be further optimized, for example, via inclusion of data from exome sequencing. Furthermore, we used a straightforward method of linking genetic variants to genes based only on proximity; thus, the longer-range effects of variants on genes were omitted from our analyses. Finally, to the extent that the central assumption of our analysis method—that some cell types make greater relative use of schizophrenia-associated genes than other cell types—is incorrect, cell type associations may be incorrect.

In conclusion, the results reported in this article are possible thanks to a combination of helpful organization in nature (the genome as a catalog of biological elements) and human technology (modern advances that allow us to query the genome, in both RNA and DNA forms, in great detail). Together, these results provide an updated framework for understanding the cellular basis of psychiatric disorders. Given the detailed molecular information available about each cell type (for example, receptors, neurotransmitters, neuropeptides), this work also suggests a roadmap toward previously unknown therapeutics, better use of existing medications and truly personalized medicine.

## Methods

This work complies with all relevant ethical regulations and was approved by Stanford University’s Institutional Review Board.

### Selecting the comparison phenotypes

In addition to schizophrenia, we selected four comparison phenotypes (alcohol consumed per week, sleep duration per night, multiple sclerosis and Alzheimer’s disease). These phenotypes met the following criteria: (1) they were polygenic; and (2) well-powered GWAS of those phenotypes were available (both conditions are required for this method). Regarding the psychiatric comparison phenotypes, we wanted to mitigate concerns about the reliability of psychiatric diagnoses. (Limitations notwithstanding, psychiatric diagnoses are moderately reliably diagnosed across time and raters. Furthermore, unbiased clustering approaches applied at the symptom level and genetic data support the utility and validity of such diagnoses.) Therefore, we chose alcohol consumed per week and sleep duration per night because they are readily quantifiable (albeit noisily). The two neurological phenotypes (multiple sclerosis and Alzheimer’s disease) were selected because they have considerably different etiology from psychiatric disorders^47^; consequently, they provided an opportunity to observe greater contrasts among this set of five brain-related phenotypes. The GWAS results used in this study were from the best-powered GWAS of these phenotypes available at the time of the analysis (November 2022 to July 2023). The relevant sample sizes, number of identified loci and source publications were as follows: schizophrenia^6^ (*n* = 320,404, loci *n* = 287); alcohol per week (*n* = 2,428,851, loci *n* = 501); sleep duration per night^17^(*n* = 446,118, loci *n* = 78); MS^18^ (*n* = 41,505, loci *n* = 233) and AD^19^ (*n* = 788,989, loci *n* = 75). Given that well-powered GWAS are required for these analyses, we used only European ancestry samples as adequately powered GWAS are not available for any other ancestry, for all these phenotypes. Additional psychiatric phenotypes analyzed were from the following publications: depression^48^; bipolar disorder^49^; anorexia^50^; alcohol use disorders^51^; PTSD^52^; autism^53^ and ADHD^54^.

### Gene expression data

The gene expression data from ref. ^10^ are publicly available. Briefly, the authors sampled 3,369,219 nuclei from 105 different dissections in three postmortem brain donors using high-throughput 10X Chromium snRNA-seq. The nuclei were then computationally clustered into 31 superclusters and 461 clusters. The 461 clusters are referred to as cell types in our study. For data processing, we first applied a transformation to the single-cell expression data to compress the scale and reduce outliers (as is typical for such data, we used ln(1 + *x*)), and obtained the mean-transformed expression for each gene in each cell type. We only kept protein-coding genes from the NCBI and removed unexpressed genes, genes with nonunique names and genes in the major histocompatibility complex (MHC) region (chromosome 6, base positions 25000000–34000000). To use MAGMA, we mapped Ensemble IDs to Entrez gene IDs with the Genome-wide Association for Human package (org.Hs.eg.db) in Bioconductor (v.3.12.0). Genes without unique Ensemble-Entrez mappings were removed. A metric of gene expression specificity was then calculated by dividing the transformed expression of a gene in a cell type by the sum of the transformed expression of that gene across all cell types, yielding a value between 0 and 1 that characterizes the extent to which a particular gene is expressed in a particular cell type. For example, in hippocampal neuron type 202, 12% of the total transformed gene expression of the *GUCA2A* gene is in this cell type, so the specificity score for *GUCA2A* in cell type 202 is 0.12. If a hypothetical gene were completely evenly expressed across all cell types, then the specificity score for that gene, in all cell types, would be 1/461 = 0.0022.

### MAGMA overview

MAGMA (v.1.10) is software designed for gene and gene set analysis of GWAS data; it has been extensively tested to ensure appropriate control of type I errors and adjustment for potentially confounding variables^15,55^. MAGMA uses a regression framework and a two-stage procedure to test for associations, first calculating gene-level *P* values and then using those gene-level *P* values to compute *P* values for the collection of genes. Collection of genes can either be analyzed as gene sets (using binary coding of genes that are in or out of the set) or as ‘gene properties’, meaning quantitative values assigned to all genes, as we have in this study (that is, specificity values for each gene in each cell type). We used the gene property analysis rather than arbitrarily imposing a threshold on the specificity scores to define a gene set for each cell type. We also used the optional third stage, conditional analysis, to specify likely independent associations among all significant associations.

We had a two-part rationale for using the directional (one-sided) test for association: (1) we followed previous examples^13,14^; and (2) our premise is that schizophrenia-associated cell types preferentially use schizophrenia-associated genes, and thus higher specificity (in a given cell type) is positively correlated with schizophrenia association, hence the directional hypothesis in our one-sided test. In contrast, a negative correlation seems much less plausible. This would mean that cell types that do not use schizophrenia-associated genes (or use them less than average, at least) are not schizophrenia-associated. Data distributions were assumed to be normal but this was not formally tested.

Our rationale for excluding the MHC region is that this region has high LD over a long portion of chromosome 6, leading to uncertainty about which genes account for associations in this region. While not the only region in the human genome with notably long-range LD, the MHC region is the most extreme example and it is also the strongest common variant association with schizophrenia. To avoid errors that this might introduce, we adopted a conservative approach by excluding the MHC region. This means that, for phenotypes with sufficiently strong MHC associations, cell types that preferentially use genes in the MHC region may have less significant results than they would if the MHC were retained. The X and Y chromosomes were also omitted because the GWAS datasets used in this study did not include these chromosomes.

### MAGMA gene-level analysis

We first used MAGMA to map each single-nucleotide polymorphism (SNP) to a gene if the SNP was located within 35 kilobases (kb) upstream to 10 kb downstream of that gene. We then used MAGMA’s SNP-wise mean (snp-wise=mean) model to conduct gene analysis while adjusting for LD. The LD data were from the European ancestry panel of phase 3 of the 1000 Genomes project^56^. In gene analysis, the test statistic of a gene was calculated as the sum of squared SNP *z*-statistics, where *z*-statistics were the probit transformation of SNP *P* values from the GWAS. Because the test statistic for each gene followed a mixture of independent ${{\rm{\chi }}}_{1}^{2}$ distributions under the null hypothesis, we calculated the gene *P* values accordingly (each representing the association between a phenotype and a gene).

### MAGMA gene property analysis

The MAGMA gene property analysis represents the association that a gene has with a given phenotype as a *z*-score ${z}_{\mathrm{g}}=\text{probit}\left(1-{{P}_{\mathrm{g}}}\right)$, where *P*_g_ is the *P* value of a given gene from the gene analysis step in MAGMA. Per MAGMA default, we truncated *z*-scores that were three s.d. below or six s.d. above the mean to prevent outliers from biasing the analysis results. We then conducted the gene property analysis using a multiple linear regression model:

1 $$Z={{{\beta }}}_{0}+{P}_{c}{{{\beta }}}_{1}+C{{{\beta }}}_{2}+\varepsilon$$

where $Z$ is the aforementioned *z*-score of each gene, *P*_*c*_ contains the specificity of each gene in a given cell type *c*, *C* represents the covariates and *ε* is modeled as a multivariate normal accounting for the LD between genes. Per MAGMA default, specificity values were truncated if they were five s.d. from the mean. In our analysis, covariates were gene size, gene density, sample size, inverse mean minor allele count and their log values. Lastly, we conducted a one-directional test of the coefficient *β*_1_ as a test of the association of each cell type with each phenotype. For each phenotype, 461 cell types were tested.

The MAGMA development team conducted a type I error simulation for the gene analysis stage in their v.1.08 documentation, which demonstrated well-controlled type I errors. To further test this with MAGMA’s gene property analysis, we used a simulation approach based on that of refs. ^13,14^. Specifically, we randomly permuted gene labels in the schizophrenia gene analysis result file 1,000 times and examined cell type associations with schizophrenia. Across 461 cell types in 1,000 permutations (that is, 461,000 simulation instances), we found fewer significant results than expected by chance at *P* < 0.05 (13,565 compared to 23,050 significant results expected by chance). We also found fewer significant results than expected by chance at our Bonferroni significance threshold of *P* < 0.05/461 = 0.0001 (that is five versus 50 expected by chance). These simulations demonstrate appropriate control of type I errors for MAGMA’s gene property analysis.

### MAGMA conditional analysis

To specify probably independent signals from among all significant results (that is, all significant cell types for each phenotype), we conducted pairwise conditional analyses using MAGMA. We used the following linear regression model:

2 $$Z={{{\beta }}}_{0}^{{\prime} }+{P}_{{c}_{1}}{{{\beta }}}_{1}^{{\prime} }+{P}_{{c}_{2}}{{{\beta }}}_{2}^{{\prime} }+C{{{\beta }}}_{3}^{{\prime} }+\varepsilon$$

This is the same model as the one from the gene property analysis except that this model includes two cell types of interest. We then conducted forward stepwise selection as detailed in ref. ^12^ to arrive at a set of relatively independent significant cell types. For cell types *c*_1_ and *c*_2_, let us denote the *P* value associated with ${{{\beta }}}_{1}^{{\prime} }$ in equation (2) be ${P}_{{c}_{1},{c}_{2}}$ and the one associated with ${{{\beta }}}_{2}^{{\prime} }$ be ${P}_{{c}_{2},{c}_{1}}$. We also denoted the marginal *P* values associated with the respective cell types from the gene property analysis as ${P}_{{c}_{1}}$ and ${P}_{{c}_{2}}$. We defined proportional significance, which portrays the remaining significance of a cell type ${c}_{1}$ after conditioning on *c*_2_ as ${{PS}}_{{c}_{1},{c}_{2}}$ such that ${{PS}}_{{c}_{1},{c}_{2}}=\frac{-\log ({P}_{{c}_{1},{c}_{2}})}{-\log ({P}_{{c}_{1}})}$. In the forward stepwise selection, the set of independent significant cell types (denoted as *S*) initially only contained the most marginally significant cell type. The next most significant cell type *c* was added to the set *S* in succession only if it satisfied two scenarios: first, if both ${{PS}}_{c,s}$ ${{PS}}_{s,c}\ge 0.8$ for all $s\in S$, the associations of cell type $c$ and any $s\in S$ with a given phenotype were considered independent. Second, if ${0.5\le {PS}}_{c,s} < 0.8,{0.5\le {PS}}_{s,c} < 0.8$, and ${P}_{c}\le 0.05$ for all $s\in S$, the associations of cell type $c$ and any $s\in S$ were only partially explained by each other, while most of the signals were independent. Cell types not included in set *S* can still have an important role in the etiology of a phenotype; however, the selection procedure excluded them because their association cannot be distinguished from the association of cell types in *S*. In some rare cases, a cell type with a lower marginal significance can have a higher conditional significance. When ${{PS}}_{{c}_{1},{c}_{2}} < 0.2$ yet ${{PS}}_{{c}_{2},{c}_{1}}\ge 0.2$ for cell type *c*_1_ and *c*_2_, where ${P}_{{c}_{1}} < {P}_{{c}_{2}}$, the order of the selection process was reversed for the two cell types.

### LDSC-SEG analysis

For each cell type, we obtained a genome annotation by taking the genes with top 10% specificity and added 100-kb windows around each gene. We then tested whether the per-SNP heritability was enriched for each annotation with LDSC to assess associations between phenotypes and cell types (see ref. ^21^ for the details).

### Exploratory analysis

The exploratory analysis of drug targets used WebGestalt^57^ to test for the enrichment of drug targets (for specific drugs or classes of drugs) among the top 200 most specific genes for each of the ten relatively independent, significant cell types for schizophrenia. We also investigated the biological processes, cellular components and molecular functions of individual cell types to the extent possible, given current knowledge about these categories. In this exploratory analysis, we used WebGestalt^57^ to assess the overrepresentation of the top 200 genes (for individual cell types) in GO categories for biological processes, cellular components and molecular functions. The results are provided in Supplementary Tables 18–22.

### Considerations regarding anatomical annotations

**Extended Data Fig. 10:**
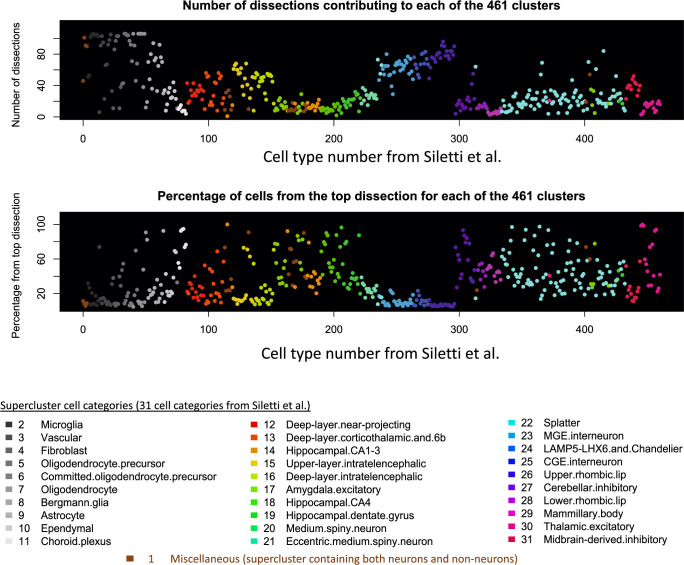
Information about the number of dissections contributing to each of the 461 cell types. For example, interneurons (cell types #236-296), shown in blue shades, are among the cell types that were found in the largest number of dissections (top). For these interneurons, no single dissection accounted for >25% of any cell type, and for most the top contributing dissection accounted for <10% of the cells in that cell type (bottom). The number of contributing dissections for these widely-distributed interneuron cell types was 56-84 (out of the 105 possible dissections performed across human brain), as described in Siletti et al.^10^.

Throughout this article, we refer to the anatomical locations of cell types. These locations were reported in ref. ^10^, where procedures involved sampling from 105 separate human brain regions (dissections) and to which each of the 3,369,219 cells in that study can be traced. However, it is rare for any of the 461 cell types to be exclusively from any one dissection or brain region. Indeed, one goal of such transcriptomic surveys is to reveal the ways in which transcriptomically similar cells are found in different areas of the brain. Extended Data Fig. 10 summarizes the information about the dissections contributing to each cell type. For example, interneurons, which are inhibitory neurons found widely across the cortex (cell types nos. 236–296 in ref. ^10^) are derived from dozens of cortical (and even some subcortical) regions. Accordingly, the single dissection contributing the most cells to any of the interneuron clusters typically accounts for less than 10% of cells in the cluster. Conversely, cell type no. 202 is from just two dissections, both in the hippocampus. For brevity, in the article we refer to cell types as hippocampal or amygdala, and so on when cell types are derived primarily from that brain region (that is more than 50%, but typically much higher). Precise information about the dissections accounting for cells in each cell type is given in Supplementary Table 1, which provides the total number of cells in each cluster and the percentage of cells in that cluster that came from each contributing dissection.

### Cell types and cell nomenclature

The nomenclature for cell types will continue to evolve as the naming and categorization of cell types continues. For clarity, we did not address the issue of continuous gradients of difference across cell types, but this will be an important area of investigation for future studies.

### Statistics and reproducibility

We explicitly addressed statistical issues throughout the article and especially in Fig. 5, Extended Data Fig. 5 and in the accompanying text. Furthermore, in all instances we used the largest sample sizes available for GWAS and snRNA-seq datasets (for phenotypes and human brain, respectively). We had no control over sample sizes. No data were excluded from the analyses. Finally, the data used were not from clinical trials (or experiments); rather, all data came from studies that were observational in nature. Thus, randomization could not be performed.

### Reporting summary

Further information on research design is available in the Nature Portfolio Reporting Summary linked to this article.

## Online content

Any methods, additional references, Nature Portfolio reporting summaries, source data, extended data, supplementary information, acknowledgements, peer review information; details of author contributions and competing interests; and statements of data and code availability are available at 10.1038/s41593-024-01834-w.

## Supplementary information

Reporting Summary

Supplementary Tables Supplementary Tables 1–17.

Supplementary Table 18 WebGestalt enrichment tables for GO terms for the schizophrenia GWAS.

Supplementary Table 19 WebGestalt enrichment tables for GO terms for the alcohol per week GWAS.

Supplementary Table 20 WebGestalt enrichment tables for GO terms for the sleep duration per night GWAS.

Supplementary Table 21 WebGestalt enrichment tables for GO terms for the MS GWAS.

Supplementary Table 22 WebGestalt enrichment tables for GO terms for the AD GWAS.

Supplementary Table 23 LDSC-SEG results.

## Data Availability

All data used in this report are publicly available. The GWAS datasets for the psychiatric phenotypes are from the Psychiatric Genomics Consortium^15^. Data for the alcohol consumed per week^16^, sleep duration per night^17^, multiple sclerosis ^18^ and Alzheimer’s disease^19^ are available in the relevant publications. The snRNA-seq dataset used in this study can be downloaded as described in ref. ^10^.

## Code availability

The code used for our implementation of the MAGMA analysis pipeline is available on GitHub (https://github.com/Integrative-Mental-Health-Lab/linking_cell_types_to_brain_phenotypes).
