# Supplementary material for: Mapping the cellular etiology of schizophrenia and complex brain phenotypes
Source: Nat Neurosci. 2025 Jan 20;28(2):248–58. doi: 10.1038/s41593-024-01834-w (PMC11802450; doi:10.1038/s41593-024-01834-w)
Supplement: Supplementary file 1 — Reporting Summary [file 41593_2024_1834_MOESM1_ESM.pdf]

nature portfolio

Corresponding author(s): Laramie Duncan

Last updated by author(s): Oct 7, 2024

Reporting Summary

Nature Portfolio wishes to improve the reproducibility of the work that we publish. This form provides structure for consistency and transparency in reporting. For further information on Nature Portfolio policies, see our Editorial Policies and the Editorial Policy Checklist.

Statistics

For all statistical analyses, confirm that the following items are present in the figure legend, table legend, main text, or Methods section.

n/a	Confirmed	
☐	☒	The exact sample size (*n*) for each experimental group/condition, given as a discrete number and unit of measurement
☒	☐	A statement on whether measurements were taken from distinct samples or whether the same sample was measured repeatedly
☐	☒	The statistical test(s) used AND whether they are one- or two-sided<br>*Only common tests should be described solely by name; describe more complex techniques in the Methods section.*
☐	☒	A description of all covariates tested
☐	☒	A description of any assumptions or corrections, such as tests of normality and adjustment for multiple comparisons
☐	☒	A full description of the statistical parameters including central tendency (e.g. means) or other basic estimates (e.g. regression coefficient) AND variation (e.g. standard deviation) or associated estimates of uncertainty (e.g. confidence intervals)
☐	☒	For null hypothesis testing, the test statistic (e.g. *F*, *t*, *r*) with confidence intervals, effect sizes, degrees of freedom and *P* value noted<br>*Give P values as exact values whenever suitable.*
☒	☐	For Bayesian analysis, information on the choice of priors and Markov chain Monte Carlo settings
☒	☐	For hierarchical and complex designs, identification of the appropriate level for tests and full reporting of outcomes
☒	☐	Estimates of effect sizes (e.g. Cohen's *d*, Pearson's *r*), indicating how they were calculated

Our web collection on statistics for biologists contains articles on many of the points above.

Software and code

Policy information about availability of computer code

Data collection

No new data were collected, and therefore no software was used for data collection.

Data analysis

Analyses used currently available software and all parameters used are stipulated in the manuscript as follows.

MAGMA overview

MAGMA (v1.10) is software designed for gene and gene set analysis of GWAS data, and it has been extensively tested to ensure appropriate control of type I errors and adjustment for potentially confounding variables26,69. MAGMA uses a regression framework and employs a two-stage procedure to test for associations, first calculating gene level p-values and then using those gene level p-values to compute p-values for collections of genes. Collections of genes can either be analyzed as gene sets (using binary coding of genes that are in or out of the set) or as 'gene properties' meaning quantitative values assigned to all genes, as we have here (i.e., specificity values for each gene, in each cell type). We employed the gene property analysis rather than arbitrarily imposing a threshold on the specificity scores to define a gene sets for each cell type. We also used the optional third stage, conditional analysis, to specify likely independent associations among all significant associations.

We had a two-part rationale for using the directional (one-sided) test for association: 1) We followed prior examples24,25, and 2) our premise is that schizophrenia associated cell types preferentially use schizophrenia associated genes, and thus higher specificity (in a given cell type) is positively correlated with schizophrenia association. This is the directional hypothesis in our one-sided test. In contrast, a negative correlation seems much less plausible. This would mean that cell types that do not use schizophrenia associated genes (or use them less than average, at least) are schizophrenia associated.

Our rational for excluding the major histocompatibility (MHC) region is that this region has high linkage disequilibrium over a long portion of chromosome 6, leading to uncertainty about which gene(s) account for associations in this region. While not the only region in the human genome with notably long-range linkage disequilibrium, the MHC region is the most extreme example, and it is also the strongest common variant association with schizophrenia. To avoid errors that this might introduce, we adopted a conservative approach by excluding the MHC region. This means that, for phenotypes with sufficiently strong MHC associations, cell types that preferentially use genes in the MHC region may have less significant results than they would if the MHC were retained. The X and Y chromosomes were also omitted because the GWAS datasets used here did not include these chromosomes.

MAGMA gene level analysis

We first used MAGMA to map each SNP to a gene if the SNP was located within 35 kilobases (kb) upstream to 10 kb downstream of that gene. We then used MAGMA's SNP-wise mean (snp-wise=mean) model to conduct gene analysis while adjusting for linkage disequilibrium (LD). LD data was from the European ancestry panel of 1000 Genomes phase 370. In gene analysis, the test statistic of a gene was calculated as the sum of squared SNP z-statistics, where z-statistics were the probit transformation of SNP p-values from GWAS. Because the test statistic for each gene followed a mixture of independent χ_1^2 distributions under the null hypothesis, we calculated gene p-values (each representing the association between a phenotype and a gene) accordingly.

MAGMA gene property analysis

MAGMA's gene property analysis represents the association that a gene has with a given phenotype as a z-score $Z_g=\text{"probit"}\ (1-Pg)$ where P_g is the p-value of a given gene from the gene analysis step in MAGMA. Per MAGMA default, we truncated z-scores that were 3 standard deviations below or 6 standard deviations above the mean to prevent outliers from biasing analysis results. We then conducted the gene property analysis via a linear regression model $Z=\beta_0+P_c \beta_1+C\beta_2+\varepsilon$ (eq.1), where Z is the aforementioned z-scores of each gene, P_c is the specificity of each gene in a given cell c, C represents the covariates, and ε is modeled as a multivariate normal accounting for the LD between genes. Per MAGMA default, specificity values were truncated if they were 5 standard deviations from the mean. In our analysis, covariates were gene size, gene density, sample size, inverse mean minor allele count, and their log values. Lastly, we conducted a one-directional test of the coefficient β_1 as a test of the association of each cell type with each phenotype. For each phenotype (e.g., schizophrenia), this analysis was run 461 times (i.e., once for each cell type).

The MAGMA development team has conducted a Type I error simulation for the gene analysis stage in their version 1.08 documentation, which demonstrates well-controlled Type I error. For gene property analysis, we used a simulation approach based on that of Skene et al. and Bryois et al.24,25 Specifically, we randomly permuted gene labels in the schizophrenia gene analysis result file 1000 times and examined cell type associations with schizophrenia. Across 461 cell types in 1000 permutations (i.e., 461,000 simulation instances), we found 13,565 significant results at $P<0.05$ (23,050 significant results expected by chance) and 5 significant results at $P<0.05/461\tilde{=}0.0001$ (50 expected by chance). This simulation demonstrates appropriate control of Type I errors (Fig. 1) for MAGMA's gene property analysis (see Supplementary Fig. 11).

MAGMA conditional analysis

To specify likely independent signals from among all significant results (i.e. all significant cell types for each phenotype), we conducted pairwise conditional analyses using MAGMA. Here, we used the linear regression model $Z=\beta_0'+P_{c_1} \beta_1'+P_{c_2} \beta_2'+C\beta_3'+\varepsilon$ (eq.2), which is the same as the one from gene property analysis except that the model here includes two cell types of interest. We then conducted forward stepwise selection as detailed in Watanabe et al.23 to arrive at a set of "independent significant" cell types. For cell type c_1 and c_2, let us denote the p-value associated with β_1' in eq.2 be p_{c_1,c_2} and the one associated with β_2' be p_{c_2,c_1}. We also denote the marginal p-values associated with the respective cell types from gene property analysis be p_{c_1} and p_{c_2}. We define proportional significance, which portrays the remaining significance of a cell type c_1 after conditioning on c_2, as ?PS?_(c_1,c_2) such that ?PS?_(c_1,c_2)=(-log(p_(c_1,c_2)))/(-log?(p?_(c_1))). In forward stepwise selection, the set of independent significant cell types (denoted as S) initially only contained the most marginally significant cell type. The next most significant cell type c was added to the set S in succession only if it satisfied two scenarios: First, if both ?PS?_(c,s),? PS?_(s,c) ≥0.8 for all s ?S, the associations of cell type c and any s ?S with a given phenotype were considered independent. Second, if ?0.5≤PS?_(c,s)<0.8 ,?0.5≤ PS?_(s,c)<0.8, and $p_c \le 0.05$ for all s ?S, the associations of cell type c and any s ?S were only partially explained by each other, while the majority of the signals were independent. Cell types not included in the set S can still play an important role in the etiology of a phenotype; however, the selection procedure excluded them because their association cannot be distinguished from the association of cell types in S. Note that in some rare cases, cell type with a lower marginal significance can have a higher conditional significance. When ?PS?_(c_1,c_2)<0.2 yet ?PS?_(c_2,c_1) ≥0.2 for cell type c_1 and c_2 where $p_{c_1}<p_{c_2}$, the order of the selection process was reversed for the two cell types.

LDSC-SEG analysis
For each cell type, we obtained a genome annotation by taking the genes with top 10% specificity and added 100-kb windows around each gene. We then tested whether the per-SNP heritability was enriched for each annotation with linkage disequilibrium score regression to assess associations between phenotypes and cell types. See Finucane et al.38 for details.

Code availability
Code used in this study is available at https://github.com/Integrative-Mental-Health-Lab/linking_cell_types_to_brain_phenotypes

For manuscripts utilizing custom algorithms or software that are central to the research but not yet described in published literature, software must be made available to editors and reviewers. We strongly encourage code deposition in a community repository (e.g. GitHub). See the Nature Portfolio guidelines for submitting code & software for further information.

Data

Policy information about availability of data

All manuscripts must include a data availability statement. This statement should provide the following information, where applicable:

- Accession codes, unique identifiers, or web links for publicly available datasets
- A description of any restrictions on data availability
- For clinical datasets or third party data, please ensure that the statement adheres to our policy

DATA AVAILABILITY
All data used in this report are publicly available. The GWAS datasets for psychiatric phenotypes are from the Psychiatric Genomics Consortium, and the relevant download page is: 15. Data for alcohol consumed per week16, sleep per night17, multiple sclerosis18, and Alzheimer's disease19 are available as described in the relevant publications. The snRNAseq dataset used here is downloadable as described in Siletti et al.10.

Research involving human participants, their data, or biological material

Policy information about studies with human participants or human data. See also policy information about sex, gender (identity/presentation), and sexual orientation and race, ethnicity and racism.

Reporting on sex and gender	Statistics and Reproducibility<br>Per journal requirements, we here address three prespecified questions regarding statistics and reproducibility. Readers will have noted that we explicitly addressed statistical issues throughout the manuscript and especially in Figure 5, Extended Data Figure 5, and in the accompanying text. The prespecified questions are as follows: 1) How were the sample sizes chosen? in all instances we used the largest sample sizes available for GWAS and snRNAseq datasets (for phenotypes and human brain, respectively). We had no control over samples sizes. 2) Was any data excluded? No data were excluded from the analyses. 3) Was randomization or blinding used? No, the data used here were not from clinical trials (nor experiments), but rather all data came from studies that were observational in nature and thus randomization could not be performed.
Reporting on race, ethnicity, or other socially relevant groupings	As noted in the limitations section of the report, we were only able to use data fromEuropean ancestry participants. For these datasets, European ancestry was defined using genomic information in contributing studies.
Population characteristics	This is a secondary data analysis using multiple prior large-scale studies of adults, as specified in the manuscript. Please note that for just one of the GWAS studies used here, specifying population information requires dozens of pages of supplmentary material, and thus It is not realistic for us to provide detailed information about the samples in this manuscript.
Recruitment	N/A
Ethics oversight	N/A

Note that full information on the approval of the study protocol must also be provided in the manuscript.

Field-specific reporting

Please select the one below that is the best fit for your research. If you are not sure, read the appropriate sections before making your selection.

☒ Life sciences ☐ Behavioural & social sciences ☐ Ecological, evolutionary & environmental sciences

For a reference copy of the document with all sections, see nature.com/documents/nr-reporting-summary-flat.pdf

Life sciences study design

All studies must disclose on these points even when the disclosure is negative.

Sample size	Sample sizes were not under our control. We used pre-existing data. We did, however, demonstrate the implications of different sample sizes on statistical power in Figure 5.
Data exclusions	No data were excluded.
Replication	To the extent possible, we explored consistency of results when sample sizes for people were smaller (Figure 5) and when the number of cells per cell type was smaller (Supplementary Figure 5).
Randomization	N/A as per the 'Statistics and Reproducibility' section of the manuscript: "...3) Was randomization or blinding used? No, the data used here were not from clinical trials (nor experiments), but rather all data came from studies that were observational in nature and thus randomization could not be performed."

Blinding

N/A as per the 'Statistics and Reproducibility' section of the manuscript: "...3) Was randomization or blinding used? No, the data used here were not from clinical trials (nor experiments), but rather all data came from studies that were observational in nature and thus randomization could not be performed."

Reporting for specific materials, systems and methods

We require information from authors about some types of materials, experimental systems and methods used in many studies. Here, indicate whether each material, system or method listed is relevant to your study. If you are not sure if a list item applies to your research, read the appropriate section before selecting a response.

Materials & experimental systems

n/a	Involved in the study
☒	☐ Antibodies
☒	☐ Eukaryotic cell lines
☒	☐ Palaeontology and archaeology
☒	☐ Animals and other organisms
☒	☐ Clinical data
☒	☐ Dual use research of concern
☒	☐ Plants

Methods

n/a	Involved in the study
☒	☐ ChIP-seq
☒	☐ Flow cytometry
☒	☐ MRI-based neuroimaging

Plants

Seed stocks

N/A

Novel plant genotypes

N/A

Authentication

N/A
